# Cerebral salt wasting syndrome due to tuberculous meningitis; a case report

**DOI:** 10.15171/jrip.2016.12

**Published:** 2016-02-20

**Authors:** Syed Ahmad, Zain Majid, Mehwish Mehdi, Muhammed Mubarak

**Affiliations:** ^1^Department of Internal Medicine, Sindh Institute of Urology and Transplantation, Karachi, Pakistan; ^2^Department of Histopathology, Sindh Institute of Urology and Transplantation, Karachi, Pakistan

**Keywords:** Cerebral salt wasting syndrome, Hyponatremia, Tuberculosis meningitis, SIADH

## Abstract

A 58-year-old male presented with fever, nausea, and vomiting since 15 days along with irritability and confusion since 5 days. His laboratory reports showed low serum sodium, serum osmolality and uric acid. Computerized tomography (CT) scan of brain revealed age-related changes. While on lumbar puncture (LP) and cerebrospinal fluid (CSF) examination, CSF protein, lactate dehydrogenase (LDH) and total leukocyte count (predominant lymphocytes) were all increased. On his 14th day of admission, his serum sodium was 116 mEq/l and he had a high urine output. Fluid restriction was tried in order to rule out syndrome of inappropriate antidiuretic hormone secretion (SIADH) but the patient did not respond to it. Keeping in view the above findings, a final diagnosis of tuberculous meningitis leading to cerebral salt wasting syndrome was made. The patient was started on 3% hypertonic saline, mineralocorticoids and anti-tuberculous therapy (ATT), to which he responded favorably and was later discharged.

**Implication for health policy/practice/research/medical education:**

Cerebral salt wasting syndrome is one of the under-recognized causes of hyponatremia and is often confused with the syndrome of inappropriate antidiuretic hormone (SIADH) secretion. It is essential to differentiate the syndrome from SIADH as the management of both conditions is drastically different but their presenting features overlap.

## Introduction

Cerebral salt wasting syndrome is an underreported cause of hyponatremia and is frequently confused with the syndrome of inappropriate antidiuretic hormone (SIADH) secretion. It is characterized by natriuresis, hyponatremia and volume contraction in response to some form of cerebral pathology (1). Differential diagnosis of this syndrome from SIADH is of paramount importance in managing a patient with cerebral salt wasting syndrome as the management of both conditions is drastically different but their presenting features overlap (2).

## Case Report

A 58-year-old man presented to us through the emergency department with fever, nausea and vomiting since 15 days along with irritability and confusion in his behaviour since 5 days. On admission, his laboratory workup showed a serum sodium of 130 mEq/l (reference range: 135-145 mEq/l), uric acid of 1.4 mg/dl (reference range: 3.4-7 mg/dl) while the rest of his electrolytes were normal and viral markers were negative. Serum osmolality turned out to be 240 mosm/kg (reference range: 285-295 mosm/kg) while his urine osmolality was 496 mosm/kg (reference range: 50-1200 mosm/kg) and fractional excretion of sodium was 1.2%. Chest x-ray, ultrasound abdomen and echocardiography were all unremarkable.

A week later, the patient became drowsy, with a decreased level of consciousness and a low Glasgow Coma Scale (GCS) score but was vitally stable. CT scan of brain was done, which showed diffuse mild age-related atrophy of the brain.

Later, a lumbar puncture (LP) was performed and cerebrospinal fluid (CSF) analysis showed a protein of 314 mg/dl (normal: <45 mg/dl), lactate dehydrogenase (LDH) of 63 U/ml (reference range: 2-7 U/ml), a total leukocyte count of 490 (reference range: 0-5) with 95% of lymphocytes. CSF culture revealed no growth while the fungal smear and the acid fast bacilli (AFB) PCR turned out to be negative.

Thyroid profile and serum cortisol levels were ordered and were within normal ranges.

During his admission, he developed a steady fall in serum sodium and on the 14th day of his stay, it was around 116 mEq/l with a high urine output of up to 4 litr/24-h. At this stage, his spot urinary sodium was 178 mEq/l (reference range: 20-40 mEq/l), and spot urine osmolality was 762 mosm/kg. Fluid restriction was tried to rule out SIADH secretion but the patient did not improve on it. Keeping in view the above laboratory findings, a final working diagnosis of tuberculosis meningitis and cerebral salt wasting syndrome was made.

The patient was treated with 3% hypertonic saline, mineralocorticoids and anti-tuberculous therapy (ATT), which not only increased his serum sodium to a normal level within 15 days but also improved his condition drastically and he was later discharged. Thus, a final diagnosis of tuberculous meningitis leading to cerebral salt wasting syndrome was made in this patient and the patient is on regular follow-up and doing well on ATT.

## Discussion

Cerebral salt wasting syndrome or renal salt wasting is mostly seen a few days after a brain injury, in patients with a normal thyroid and adrenal gland function, having a defective kidney sodium transport mechanism that leads to a decreased extracellular volume (1). Its incidence is underreported, but it is supposed to be one of the major causes of hyponatremia amongst the neurosurgical cases (2). Although similar in presentation to SIADH, a few factors help in distinguishing between the two; the main one being the effective arterial blood volume, which is decreased in cerebral salt wasting syndrome while increased in SIADH (3). Since the treatment of both conditions is different, an accurate diagnosis is necessary in order to save precious time, which if not taken into account, can lead to worsening of the condition (3).

The main diagnostic features of cerebral salt wasting syndrome are a brain lesion and a loss of sodium and chloride by the kidneys without having any stimuli for it (4).

Even though its cause is still not known, researchers have concluded that low sodium in patients with brain disease might be due to cerebral salt wasting syndrome (5). cerebral salt wasting syndrome can also occur without brain disease (1).

Younas et al in their study of 3 patients with cerebral salt wasting syndrome diagnosed their patients based on three parameters; fractional excretion of urinary sodium along with uric acid and a tremendously low serum uric acid (6). These three parameters were also measured in our case.

Nishio et al reported a case of cerebral salt wasting syndrome in a middle aged Japanese women who was diagnosed with limbic encephalitis and had an increased white blood cell count along with an increased protein on CSF analysis seen on LP and had presented with psychiatric issues (7).

Çelik et al reported 2 cases of cerebral salt wasting syndrome in children having status epilepticus (8).

Bettinelli et al studied 110 patients with brain disorders having cerebral salt wasting syndrome and concluded that one of the main underlying cause was found to be TB meningoencephalitis, as seen in our case too (9).

Treatment of cerebral salt wasting syndrome includes fluid along with sodium replacement, which is done via hypertonic saline (5). Hedge treated his case of cerebral salt wasting syndrome with saline and 0.2 mg per day of fludrocortisone, which not only improved the patient’s consciousness but also his sodium levels, a similar situation to our case (2).

## Conclusion

In conclusion, this case illustrates the point that patients with low serum sodium and some intracranial pathology might be suffering from cerebral salt wasting syndrome. This should also be differentiated from SIADH, as a wrong diagnosis could lead to an inappropriate treatment and might add to the morbidity of patients.

## Authors’ contribution

SA, ZM and MeM wrote the manuscript equally. MuM did the editing.

## Conflicts of interest

The authors declare that they have no conflicting interest.

## Ethical considerations

Ethical issues (including plagiarism, data fabrication, double publication) have been completely observed by the authors.

## Funding/Support

None.
